# Supplementary material for: A fault-intrusion-tolerant system and deadline-aware algorithm for scheduling scientific workflow in the cloud
Source: PeerJ Comput Sci. 2021 Nov 2;7:e747. doi: 10.7717/peerj-cs.747 (PMC8576554; doi:10.7717/peerj-cs.747)
Supplement: Supplemental Information 6 [file peerj-cs-07-747-s006.zip › Myproject1.1/docs/org/cloudbus/cloudsim/HostDynamicWorkload.html]

HostDynamicWorkload


JavaScript is disabled on your browser.


- Overview
- Package
- Class
- Use
- Tree
- Deprecated
- Index
- Help

- Prev Class
- Next Class

- Frames
- No Frames

- All Classes

- Summary:
- Nested |
- Field |
- Constr |
- Method

- Detail:
- Field |
- Constr |
- Method


org.cloudbus.cloudsim

## Class HostDynamicWorkload

- java.lang.Object
- - org.cloudbus.cloudsim.Host
  - - org.cloudbus.cloudsim.HostDynamicWorkload

- Direct Known Subclasses:
  :   PowerHost

  ---

    

  ```
  public class HostDynamicWorkload
  extends Host
  ```

  The class of a host supporting dynamic workloads and performance degradation.

  Since:
  :   CloudSim Toolkit 2.0

- - ### Constructor Summary

    Constructors

    Constructor and Description

    `HostDynamicWorkload(int id,
    RamProvisioner ramProvisioner,
    BwProvisioner bwProvisioner,
    long storage,
    java.util.List<? extends Pe> peList,
    VmScheduler vmScheduler)`

    Instantiates a new host.
  - ### Method Summary

    Methods

    | Modifier and Type | Method and Description |
    | --- | --- |
    | `void` | `addStateHistoryEntry(double time, double allocatedMips, double requestedMips, boolean isActive)` Adds the state history entry. |
    | `java.util.List<Vm>` | `getCompletedVms()` Gets the completed vms. |
    | `double` | `getMaxUtilization()` Gets the max utilization among by all PEs. |
    | `double` | `getMaxUtilizationAmongVmsPes(Vm vm)` Gets the max utilization among by all PEs allocated to the VM. |
    | `double` | `getPreviousUtilizationMips()` Gets the previous utilization mips. |
    | `double` | `getPreviousUtilizationOfCpu()` Gets the previous utilization of CPU in percentage. |
    | `java.util.List<HostStateHistoryEntry>` | `getStateHistory()` Gets the state history. |
    | `double` | `getUtilizationMips()` Gets the utilization mips. |
    | `double` | `getUtilizationOfBw()` Gets the utilization of bw. |
    | `double` | `getUtilizationOfCpu()` Get current utilization of CPU in percentage. |
    | `double` | `getUtilizationOfCpuMips()` Get current utilization of CPU in MIPS. |
    | `double` | `getUtilizationOfRam()` Gets the utilization of memory. |
    | `protected void` | `setPreviousUtilizationMips(double previousUtilizationMips)` Sets the previous utilization mips. |
    | `protected void` | `setUtilizationMips(double utilizationMips)` Sets the utilization mips. |
    | `double` | `updateVmsProcessing(double currentTime)` Requests updating of processing of cloudlets in the VMs running in this host. |

    - ### Methods inherited from class org.cloudbus.cloudsim.Host

      `addMigratingInVm, allocatePesForVm, deallocatePesForVm, getAllocatedMipsForVm, getAvailableMips, getBw, getBwProvisioner, getDatacenter, getId, getMaxAvailableMips, getNumberOfFreePes, getNumberOfPes, getPeList, getRam, getRamProvisioner, getStorage, getTotalAllocatedMipsForVm, getTotalMips, getVm, getVmList, getVmScheduler, getVmsMigratingIn, isFailed, isSuitableForVm, reallocateMigratingInVms, removeMigratingInVm, setBwProvisioner, setDatacenter, setFailed, setFailed, setId, setPeList, setPeStatus, setRamProvisioner, setStorage, setVmScheduler, vmCreate, vmDeallocate, vmDeallocateAll, vmDestroy, vmDestroyAll`
    - ### Methods inherited from class java.lang.Object

      `clone, equals, finalize, getClass, hashCode, notify, notifyAll, toString, wait, wait, wait`

- - ### Constructor Detail


    - #### HostDynamicWorkload

      ```
      public HostDynamicWorkload(int id,
                         RamProvisioner ramProvisioner,
                         BwProvisioner bwProvisioner,
                         long storage,
                         java.util.List<? extends Pe> peList,
                         VmScheduler vmScheduler)
      ```

      Instantiates a new host.

      Parameters:
      :   `id` - the id
      :   `ramProvisioner` - the ram provisioner
      :   `bwProvisioner` - the bw provisioner
      :   `storage` - the storage
      :   `peList` - the pe list
      :   `vmScheduler` - the VM scheduler
  - ### Method Detail


    - #### updateVmsProcessing

      ```
      public double updateVmsProcessing(double currentTime)
      ```

      **Description copied from class: `Host`**

      Requests updating of processing of cloudlets in the VMs running in this host.

      **Overrides:**
      :   `updateVmsProcessing` in class `Host`

      Parameters:
      :   `currentTime` - the current time

      Returns:
      :   expected time of completion of the next cloudlet in all VMs in this host.
          Double.MAX\_VALUE if there is no future events expected in this host


    - #### getCompletedVms

      ```
      public java.util.List<Vm> getCompletedVms()
      ```

      Gets the completed vms.

      Returns:
      :   the completed vms


    - #### getMaxUtilization

      ```
      public double getMaxUtilization()
      ```

      Gets the max utilization among by all PEs.

      Returns:
      :   the utilization


    - #### getMaxUtilizationAmongVmsPes

      ```
      public double getMaxUtilizationAmongVmsPes(Vm vm)
      ```

      Gets the max utilization among by all PEs allocated to the VM.

      Parameters:
      :   `vm` - the vm

      Returns:
      :   the utilization


    - #### getUtilizationOfRam

      ```
      public double getUtilizationOfRam()
      ```

      Gets the utilization of memory.

      Returns:
      :   the utilization of memory


    - #### getUtilizationOfBw

      ```
      public double getUtilizationOfBw()
      ```

      Gets the utilization of bw.

      Returns:
      :   the utilization of bw


    - #### getUtilizationOfCpu

      ```
      public double getUtilizationOfCpu()
      ```

      Get current utilization of CPU in percentage.

      Returns:
      :   current utilization of CPU in percents


    - #### getPreviousUtilizationOfCpu

      ```
      public double getPreviousUtilizationOfCpu()
      ```

      Gets the previous utilization of CPU in percentage.

      Returns:
      :   the previous utilization of cpu


    - #### getUtilizationOfCpuMips

      ```
      public double getUtilizationOfCpuMips()
      ```

      Get current utilization of CPU in MIPS.

      Returns:
      :   current utilization of CPU in MIPS


    - #### getUtilizationMips

      ```
      public double getUtilizationMips()
      ```

      Gets the utilization mips.

      Returns:
      :   the utilization mips


    - #### setUtilizationMips

      ```
      protected void setUtilizationMips(double utilizationMips)
      ```

      Sets the utilization mips.

      Parameters:
      :   `utilizationMips` - the new utilization mips


    - #### getPreviousUtilizationMips

      ```
      public double getPreviousUtilizationMips()
      ```

      Gets the previous utilization mips.

      Returns:
      :   the previous utilization mips


    - #### setPreviousUtilizationMips

      ```
      protected void setPreviousUtilizationMips(double previousUtilizationMips)
      ```

      Sets the previous utilization mips.

      Parameters:
      :   `previousUtilizationMips` - the new previous utilization mips


    - #### getStateHistory

      ```
      public java.util.List<HostStateHistoryEntry> getStateHistory()
      ```

      Gets the state history.

      Returns:
      :   the state history


    - #### addStateHistoryEntry

      ```
      public void addStateHistoryEntry(double time,
                              double allocatedMips,
                              double requestedMips,
                              boolean isActive)
      ```

      Adds the state history entry.

      Parameters:
      :   `time` - the time
      :   `allocatedMips` - the allocated mips
      :   `requestedMips` - the requested mips
      :   `isActive` - the is active


- Overview
- Package
- Class
- Use
- Tree
- Deprecated
- Index
- Help

- Prev Class
- Next Class

- Frames
- No Frames

- All Classes

- Summary:
- Nested |
- Field |
- Constr |
- Method

- Detail:
- Field |
- Constr |
- Method
